# Bone density of the axis (C2) measured using Hounsfield units of computed tomography

**DOI:** 10.1186/s13018-023-03560-8

**Published:** 2023-02-10

**Authors:** George Simion, Niklas Eckardt, Christian Senft, Falko Schwarz

**Affiliations:** 1 Department of Neurosurgery, Jena University Hospital – Friedrich Schiller University Jena, Jena, Germany; 2 Department for Radiology, Jena University Hospital – Friedrich Schiller University Jena, Jena, Germany

**Keywords:** Osteoporosis, Fracture, Bone density, Cervical spine

## Abstract

**Introduction:**

The assessment of bone density is of great importance nowadays due to the increasing age of patients. Especially in regard to the surgical stabilization of the spine, the assessment of bone density is important for therapeutic decision making. The aim of this work was to record trabecular bone density values using Hounsfield units of the second cervical vertebra.

**Material and methods:**

The study is a monocentric retrospective data analysis of 198 patients who received contrast-enhanced polytrauma computed tomography in a period of two years at a maximum care hospital. Hounsfield units were measured in three different regions within the C2: dens, transition area between dens and vertebral body and vertebral body. The measured Hounsfield units were converted into bone density values using a validated formula.

**Results:**

A total of 198 patients were included. The median bone density varied in different regions of all measured C2 vertebrae: in the dens axis, C2 transition area between dens and vertebral body, and in the vertebral body bone densities were 302.79 mg/cm^3^, 160.08 mg/cm^3^, and 240.31 mg/cm^3^, respectively. The transition area from dens axis to corpus had statistically significant lower bone density values compared to the other regions (*p* < 0.001). There was a decrease in bone density values after age 50 years in both men and women (*p* < 0.001).

**Conclusions:**

The transitional area from dens axis to corpus showed statistically significant lower bone density values compared to the adjacent regions (*p* < 0.001). This area seems to be a predilection site for fractures of the 2nd cervical vertebra, which is why special attention should be paid here in radiological diagnostics after a trauma.

## Introduction

The assessment of bone density is of great importance nowadays due to the increasing age of patients. Especially with regard to the surgical stabilization of the spinal vertebrae following trauma, the assessment of bone density is important for the therapy decision. There are already several studies in the literature showing a significant correlation between the Hounsfield units (HU) of a computed tomography and the bone density measured by DXA (Dual energy X-ray absorptiometry) [1–7].

Measuring bone density is important in the context of diagnosing osteoporosis. The clinical importance of osteoporosis refers to the increased risk of fractures [8, 9]. Osteoporosis is a disease that has received increasing attention due to the increasing age of the population. Bone density assessment is therefore very important [9]. Worldwide, osteoporosis causes more than 8.9 million fractures annually [10]. Often, the diagnosis is not made until an osteoporotic fracture occurs. In 2010, it was estimated that there were 158 million people at high risk of fracture. Due to demographic change, this number is expected to double by 2040 [11]. According to Klotzbuecher et al. [12], a loss of 10% bone mass in the vertebrae can double the risk of vertebral fractures, and a loss of 10% bone mass in the hip can similarly lead to a 2.5-fold higher risk of hip fractures. Osteoporotic fractures lead to increased morbidity and mortality [13]. It is already known that age is an independent factor in the reduction of bone density [14, 15]. Yu et. al reported a more rapid decrease in spinal bone density in women in age groups after 40–49 years compared to men [16].

For bone density measurement, DXA is the gold standard [17]. However, this examination has some disadvantages: For example, it cannot distinguish between cortical bone and cancellous bone, cannot examine specific spinal segments, and has a high cost.

There are several methods to determine bone density based on a clinical CT scan: simultaneous calibration, asynchronous calibration, internal calibration or using the HU directly. The simultaneous phantom-based calibration is used in standard quantitative CT (QCT). In this method, the bone density is calculated from the CT values using a phantom calibration containing usually hydroxyapatite which is positioned under the patient. This procedure minimizes the differences in bone density between different models of CT scanners. Asynchronous calibration does not require the presence of a phantom during CT scan. This method separates patient investigation and phantom scan. The calibration phantom can be scanned once weekly or once monthly[18]. The internal density calibration eliminates the need of a calibration phantom for opportunistic CT screening. This method uses in-scan regions of interest (ROI) in different body tissues such as subcutaneous adipose tissue and blood for calibration. Michalski et al. [19] showed in the cadaveric analyses that internal calibration performs equivalently to the phantom-based calibration. The direct use of HU is another method to determine bone density. A calibration is not performed, making this method surely the easiest to use. The direct use of HU requires a CT scanner produced by the same manufacturer or ideally the same scanner [18, 20]. Determination of HU is associated with no additional cost or additional radiation because a CT scan is usually already available after trauma or before spinal instrumentation [3]. There are already several studies in the literature showing a correlation between directly obtained HU from a CT scan and DXA and QCT values [2, 5, 21–24]. The measurement of HU is currently accepted as a good tool for measuring bone density [3]. Based on their results, Pickhardt et al. [2] claim that the cutoff for osteoporosis is 135 HU. Buenger et al. also demonstrated a significant relationship between HU measured on CT and QCT values. For both native CT examinations and contrast-enhanced CT examinations, a conversion formula for measuring bone density was proposed: QCT value = 0.7 × HU + 17.8 and QCT value = 0.71 × HU + 13.82, respectively [1, 3].

The first two vertebral bodies (C1, C2) have a significantly different structure compared to the other cervical vertebrae. C1 and C2 share about 60% of the rotational and 40% of the flexion–extension movements [25]. Fractures of C2 are the most common cervical spinal injury among elderly [26, 27]. C2 fractures can be subdivided into odontoid fractures, Hangman's fractures, and atypical fractures [28]. The odontoid type 2 fractures are the most common type of C2 fractures [27].

The aim of this work was to evaluate the trabecular bone density values using Hounsfield units of the second cervical vertebra. Furthermore, it was to be examined whether differences in bone density exist between the vertebral regions of the axis in relation to sex and age.

## Material and methods

This study is a monocentric retrospective data analysis. 198 patients who received contrast-enhanced polytrauma CT scans (256-slice Multi Detector Ct Scanner GE Healthcare Revolution; slice thickness 0.625 mm; tube spectra 80–120 kV, tube current: Smart mA 100–755, voxel size: 1,25 mm, pitch: 0.922:1, rotation time: 0.5 s, detection coverage: 80 mm) in a period between 01/01/2020 and 31/06/2021 at a maximum care hospital were included in the study. Patients were subsequently included in chronological order depending on the date of examination. Data collection was performed anonymously in an Excel spreadsheet by a single physician. Basic information (patient age, sex, examination date) and Hounsfield units and vertebral bone density of C2 were recorded. Bone density values were calculated using the formula of Buenger et al. (QCT value = 0.71 × HU + 13.82) [1]. The data were stratified by sex and decade of life. The overall study design and conduct were approved by the local ethic committee (Reg.-Nr.: 2020-2030-Daten).

Patients without age limitation who underwent contrast-enhanced polytrauma CT polytrauma were included.

Exclusion criteria were pathologies such as C2 fractures, surgery with material implantation in the upper cervical spine, signs of osteochondrosis or spondylodiscitis and artifacts due to implanted materials or other causes.

### Measurement of the hounsfield units

**Fig. 1:**
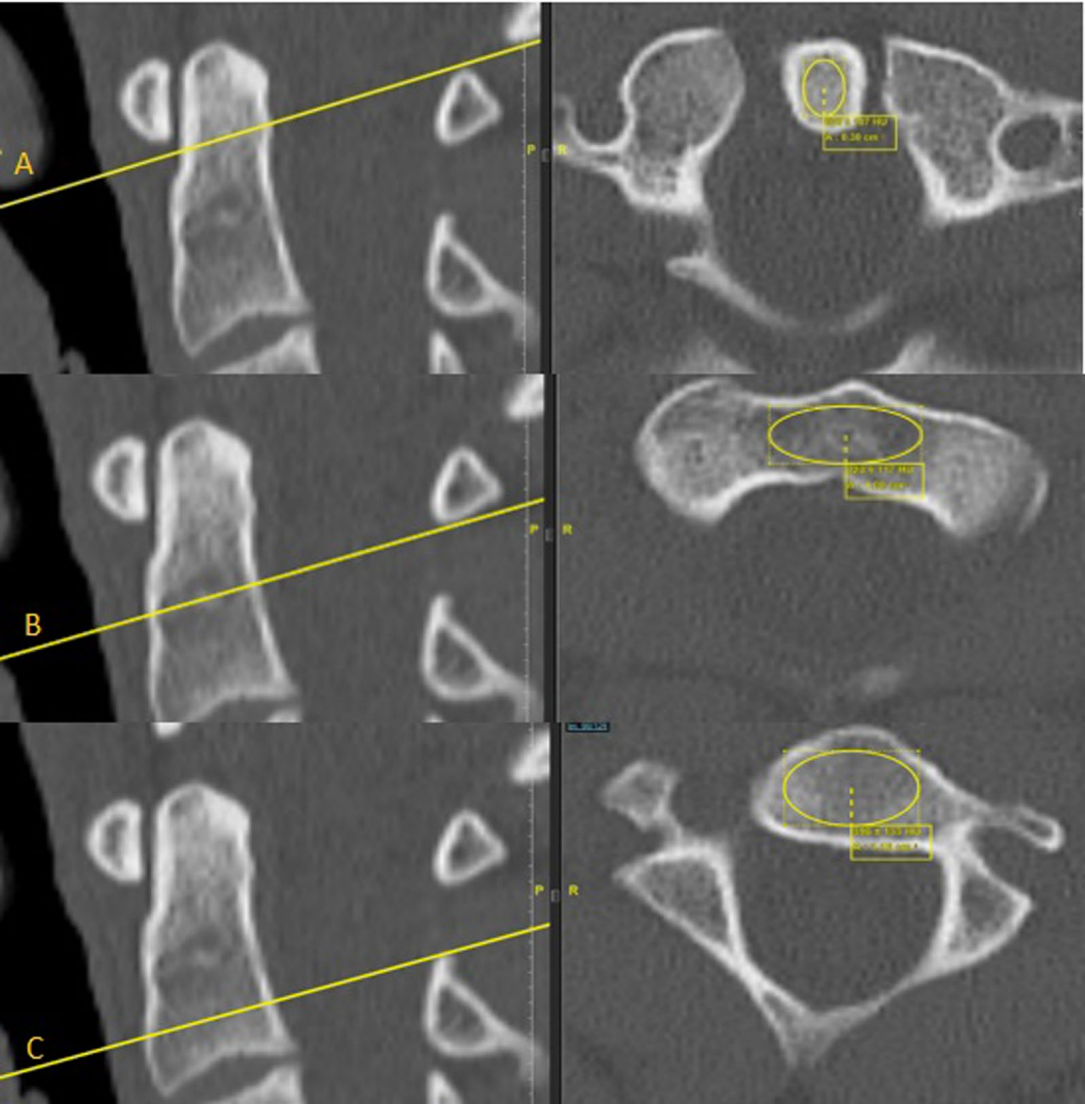
Determination of HU of the 2nd vertebra in 3 regions: Dens axis (**A**), transition area (**B**) and vertebral body (**C**) using computed tomography

Hounsfield units (HU) were recorded in the axial plane. HU were measured with an elliptical measurement field in three different localizations within the vertebral body: dens, transition area between dens and vertebral body and vertebral body. The transition zone was also localized using coronal CT imaging and was defined as the area between the dens and vertebral body of C2. In the axial plane, the "region of interest" (ROI) was chosen as large as possible, leaving out the cortical bone. Thus, only the trabecular bone was measured (Fig. 1).

The HU were measured in the Centricity Universal Viewer Zero (GE Healthcare, Chicago, USA). The measurement of HU values was always performed by the same physician.

### Statistics

The data were recorded anonymously in Microsoft Excel. Statistical analysis was performed using SPSS 26 (IBM Inc, USA). Data were grouped by sex and age (10-year intervals).

The data were not normally distributed, so nonparametric tests were used for statistical analysis. Comparison of bone density of different localizations was performed using the two-sided Friedman test. Comparison of 2 localizations was performed using the two-sided Wilcoxon test. The sex-based comparison was performed using the two-sided Mann–Whitney *U* test. Age-by-age comparison was performed using the two-sided Kruskal–Wallis test. Paired comparisons between the age groups were performed using the two-sided Mann–Whitney *U* test. The *p* value was adjusted for multiple tests using Bonferroni correction.

For all analyses, a *p* value < 0.05 was assumed to be significant.

## Results

A total of 198 patients were included (153 males and 45 females). The patients were on average 47.05 years old at the time of study (range min. 10; max. 89).

The mean and median bone density of all measured C2 vertebrae was 237.25 mg/cm^3^ and 228.47 mg/cm^3^, respectively (min. 103.99; max. 483.84 mg/cm^3^). The bone density values of each region of the axis are presented in Table 1.

**Table 1:** Bone density values in mg/cm^3^ in dens axis, transition area and corpus axis

	*N*	Mean (mg/cm^3^)	Median (mg/cm^3^)	Standard deviation	Minimum	Maximum
Dens axis	198	303.84	302.79	88.80	118.19	564.07
Transition area	198	168.72	160.08	61.65	51.45	402.19
Corpus axis	198	239.21	240.31	65.93	117.48	541.35

*C* cervical, *N* number of cases

Significant decrease in values (dens > C2 vertebral body > transitional area; *p* < 0.001)

The Shapiro-Wilks test (*p* < 0.05) and visual inspection of its histograms as well as QQ charts showed that bone density values were not normally distributed. Therefore, further statistical analysis was performed with nonparametric tests.

**Fig. 2:**
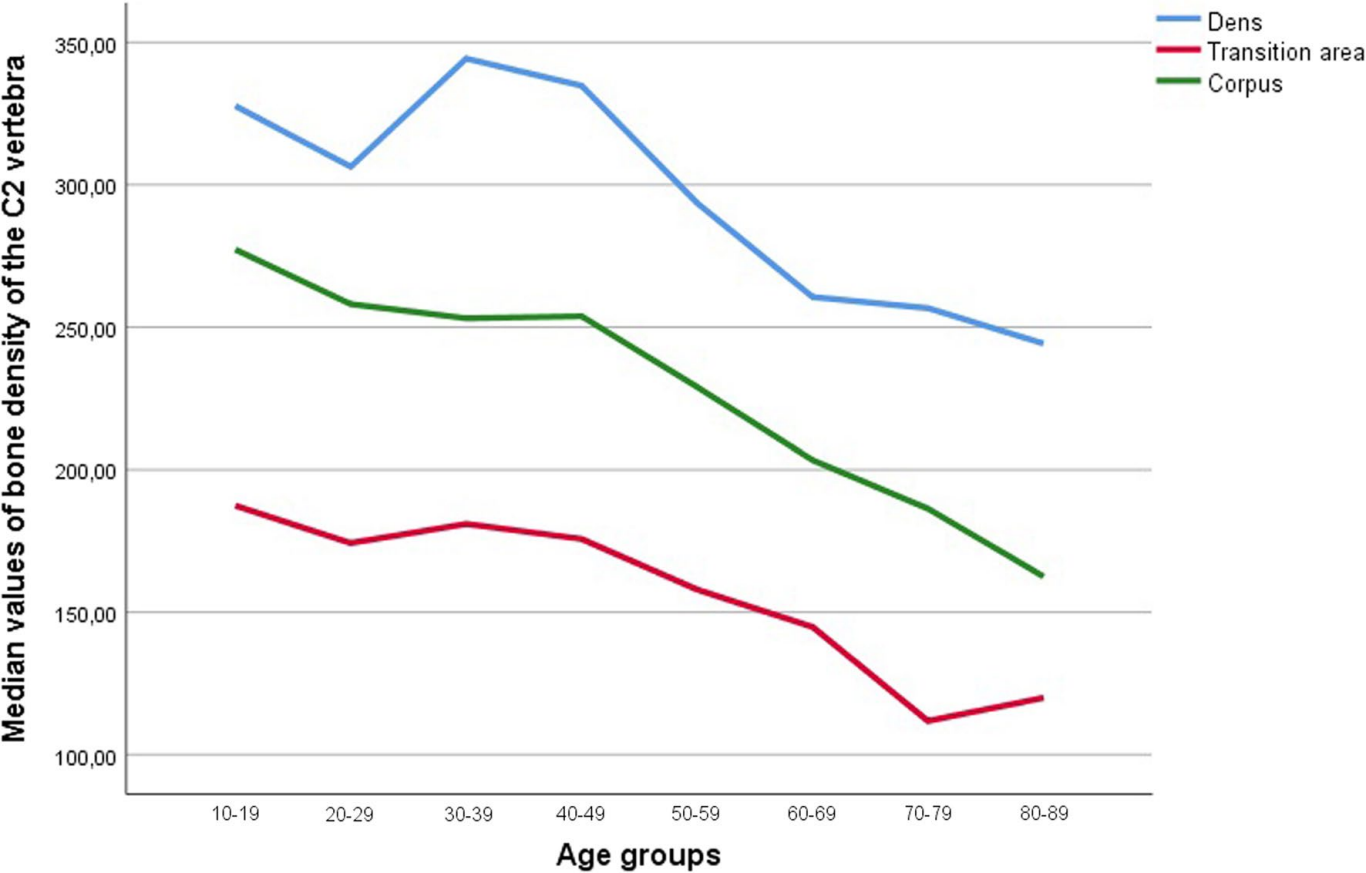
Bone density values of all patients in the area of dens axis, transition area and corpus axis according to age group

There was a statistically significant difference in bone density values in the different regions of the second cervical vertebra (*p* < 0.001). Statistically significant lower values were measured in the C2 transition area between dens and vertebral body compared to the other regions (*p* < 0.001, Table 1). The same decrease in bone density values occurred between dens axis to the corpus of C2 with a further decrease to the C2 transition area. This was also noticeable when comparing by sex (*p* < 0.001, Table 2). In Fig. 2 are shown the bone density values in the dens, transition area and vertebral body according to age group.

**Table 2:** Bone density values in mg/cm^3^ in dens axis, transition area and corpus axis in females and males

	Females			Males		
	*N*	Mean (mg/cm^3^)	Median (mg/cm^3^)	*N*	Mean (mg/cm^3^)	Median (mg/cm^3^)
Dens axis	45	288.62	270.13	153	308.32	308.47
Transition area	45	158.08	155.11	153	171.85	162.21
Corpus axis	45	237.55	231.79	153	239.69	241.73

*N* number of cases

Significant decrease in values (dens axis > corpus > transition area; *p* < 0.001)

The difference between the bone density values between males and females was not statistically significant (231.79 vs. 213.56 mg/cm^3^; *p* = 0.172).

There was a statistically significant difference between the bone density values of the different age groups (*p* < 0.001, Table 3). A reduction in bone density was observed in patients over 50 years of age, with a statistically significant difference in paired comparisons of age groups below 50 years with those above 50 years (*p* < 0.001). Similar results were seen when comparing bone density values above 50 years of age for the different regions of C2 vertebra. The decrease in values was observed in both females and males (*p* < 0.001).

**Table 3:** Bone density values in mg/cm^3^ of C2 of different age groups

Age group	*N*	Mean (mg/cm^3^)	Median (mg/cm^3^)	Standard deviation	Minimum	Maximum
10–19 years	16	272.63	271.08	50.27	201.26	411.18
20–29 years	25	253.54	241.26	51.51	185.40	364.56
30–39 years	36	260.78	253.92	62.06	126.47	392.01
40–49 years	25	267.00	245.75	73.81	145.88	447.16
50–59 years	39	228.28	221.14	62.67	126.95	483.84
60–69 years	28	212.17	212.74	54.76	131.68	337.11
70–79 years	17	186.92	187.06	54.87	103.99	305.87
80–89 years	12	182.66	176.17	54.34	117.95	265.63
Total	198	237.26	228.48	65.54	103.99	483.84

*N* number of cases

Significant decrease in bone density in paired comparisons of age groups below 50 years with those above 50 years (*p* < 0.001)

## Discussion

A decrease in bone density as well as changes in the basic structure of the bone characterizes osteoporosis. On the one hand, routine measurement of bone density can diagnose osteoporosis earlier, and on the other hand, measurement of bone density is a very important factor to be considered in spine surgery. There are a few studies in the literature describing the differences in bone density between different regions of C2 vertebra. This information may have an importance not only for osteoporosis diagnosis and spine surgery preparation, but also for medical device industry and scientists who want to study the biomechanical properties of vertebral bodies.

It is already known that contrast agent administration leads to a slight increase in HU values. On average, the differences between native and arterial phases in the study by Pompe et al. were 12 HU [29].

The main goal of our study was to measure bone density of the C2 vertebra in different regions. A strength of our study is the large number of patients. Likewise, the patients received the same examination after trauma. Contrast medium was applied to all patients before the examination of the spine. For these reasons, the values are very comparable with each other and between patients.

In our study, when examining the bone density of C2, it was noticed that there was a transitional area between the dens and the corpus, where statistically significantly lower values were detected than in the adjacent areas. This hypodense bone area, located immediately below the dens, was also described in the anatomical studies of Heggeness et al. and Kandziora et al. [30, 31]. This area is the area where the most common type of C2 fracture occurs: the type II odontoid fracture. This certainly seems to be related to the decrease in bone density in this area. The study by Lodin et al. is the only one we were able to find in which the bone density of the C2 vertebra was examined using HU. Bone density was measured preoperatively and postoperatively in patients with an odontoid fracture. A similar decrease in transitional bone density was also described in this study. It should be noted that this study involved patients who had already sustained a dens axis fracture and not healthy patients [32]. In comparison, only patients without a fracture were examined in our study.

Some studies have already compared spinal bone density by sex. Lehmann et al. described no significant difference in bone density between premenopausal women and men [33]. Similar data were also noted by Cvijetic and Korsic [34]. However, there are several studies describing higher bone density values for premenopausal women compared with men [5, 35]. Zhang et al. described greater values of bone density in women compared with men at all ages. All patients were under 59 years of age [25]. When comparing bone density by sex, we did not find a significant statistical difference in values in our study. The bone density values of all C2 vertebra regions by gender were very similar, with no significant differences.

Comparing bone density by age, in our study, we observed a significant decrease in bone density of C2 in both men and women over 50 years of age. The decrease in bone density in women can certainly be explained by postmenopausal changes and was similarly described by Lehman et al. when measuring bone density using DXA [33]. However, the authors did not observe a significant decrease in bone density with the increase in age in men. Zhang et al. described higher bone density values in women compared with men at all ages [25]. The difference between the Lehmann et al. and Zhang studies may perhaps come from the different study methods. It is well known that both cortical and trabecular bones are examined using the DXA examination. However, overall, a decrease in bone density in postmenopausal women is certainly very credible and physiologically explainable. In our study, this was also observed. One possible cause of the marked decrease in bone density in men after the age of 50 would be the decrease in testosterone levels. However, it is already known that testosterone levels only decrease at about 1 percent per year [36]. Another possible cause would be that at older ages, bones are less stressed due to limited exercise, resulting in a decrease in bone density. There are already several prospective randomized studies demonstrating the protective effect of strength training on bone density [37, 38].

Of course, our study also has limitations. The study is purely retrospective and monocentric. The CT scans were conducted in the context of a trauma. As a result, the selection of patients may naturally include subjects with pre-existing conditions. The medication that could have an influence on bone density was not recorded. Furthermore, the individual radiation dose values were not collected.

In addition, all examinations were performed with contrast medium administration. On the one hand, this is an advantage because the examinations can be compared well with each other; on the other hand, as already mentioned, the measurement of bone density is influenced by the administration of contrast medium. Another limitation of the study is that approximately three times more men than women were included in the study. This can generally be explained by a higher proportion of trauma patients being men.

Another limitation of the study is the method of determining bone density from directly measured HU on CT using the above equation of Buenger et al. [1]. Compared to the other clinically used methods for bone density determination on CT like asynchronous calibration and internal calibration, in the case of directly measured HU no calibration of the bone density values is performed. For this reason, the values obtained are dependent on the used CT scanner and may differ between CT scanners of different manufacturers. This was confirmed by a study of 67392 CT investigations obtained from four different CT scanners, showing differences in HU obtained between CT scanners [20]. In addition, the Buenger et al. equation was validated on a single CT scanner, so it is not known if it is valid on CT scanner from a different manufacturer.

In the present study, we tried to avoid possible errors coming from measurement of HU on CT investigations obtained from different scanners. All CT investigations in this study were performed on the same CT scanner.

In our study, we did not examine the inter-observer reliability, which also could influence the measurements. All HU measurements, i.e., the selection of ROI in the different areas of C2 were performed by one single person. However, in a study from 2022 also from our institution, the inter-observer reliability in direct measurement of HU of the same vertebra by four different investigators was very high [39].

## Conclusion

In summary, bone density values of the 2nd vertebra of 198 patients were determined by HU in this study. Bone density values were calculated using the formula of Buenger et. al (QCT value in mg/cm^3^ = 0.71 × HU of contrast-enhanced CT + 13.82) [1].

When bone density of the second cervical vertebra was examined, the transitional area from dens to corpus vertebra showed statistically significant lower bone density values compared to the adjacent regions (*p* < 0.001). The results are consistent with previous anatomical studies of the C2 vertebra and explain the frequency of dens fractures in this transitional region.

Bone density values generally decreased with age in all C2 regions. There was a clear decrease in values from age over 50 years, in both males and females (*p* < 0.001). The decrease in bone density in females can be explained by postmenopausal changes. A similar decrease in bone density in females was already described in the lumbar spine [33]. To our knowledge, a clear decrease in bone density of C2 vertebra in males over 50 years of age has not yet been described in the literature. Further studies examining these parameters in a different patient population are certainly needed to confirm this.

These data of bone density of C2 vertebra, among others, may be helpful to comprehensively evaluate the status of the spine and to design a better preoperative plan before instrumentation. The bone density of different vertebral bodies can be equally important to the medical device industry, which must develop instrumentation such as screws and disk replacements. These medical devices must be specifically adapted for the local anatomical conditions of different spinal regions and their bone density.

## Data Availability

The data that support the findings of this study are available on request from corresponding author, George Simion. The data are not publicly available because of their containing information that could compromise the privacy of the patients.
